# Amnion-Derived Mesenchymal Stem Cell Exosomes-Mediated Autophagy Promotes the Survival of Trophoblasts Under Hypoxia Through mTOR Pathway by the Downregulation of EZH2

**DOI:** 10.3389/fcell.2020.545852

**Published:** 2020-11-11

**Authors:** Yijing Chu, Weiping Chen, Wei Peng, Yong Liu, Lin Xu, Jianxin Zuo, Jun Zhou, Yan Zhang, Ning Zhang, Jing Li, Ling Liu, Ke Yao, Guoqiang Gao, Xiaofei Wang, Rendong Han, Chong Liu, Yan Li, Huansheng Zhou, Yuxiang Huang, Yuanhua Ye

**Affiliations:** ^1^Department of Obstetrics and Gynecology, The Affiliated Hospital of Qingdao University, Qingdao, China; ^2^Department of Anesthesiology, The Affiliated Hospital of Qingdao University, Qingdao, China; ^3^Allcare Biomedical Development, Qingdao, China

**Keywords:** chorionic villous-derived mesenchymal stem cells, trophoblasts, autophagy, EZH2, mTOR signaling

## Abstract

Human amnion-derived mesenchymal stem cells (AD-MSCs) have been reported as a promising effective treatment to repair tissue. Trophoblast dysfunction during pregnancy is significantly involved in the pathogenesis of preeclampsia (PE). To understand how AD-MSCs regulated trophoblast function, we treated trophoblasts with AD-MSC-derived exosomes under hypoxic conditions. The treatment markedly enhanced the trophoblast proliferation and autophagy. Furthermore, significant decrease of EZH2 levels and inactivation of mTOR signaling were observed in AD-MSC exosomes-treated trophoblasts. Consistent with these findings, overexpression of EZH2 activated the mTOR signaling in trophoblasts, and reduced the autophagy and survival of trophoblasts, even in the presence of AD-MSC-derived exosomes. In addition, EZH2 inhibition exhibited the same trophoblast autophagy-promoting effect as induced by AD-MSC-derived exosomes, also accompanied by the inactivation of mTOR signaling. Importantly, when EZH2 was overexpressed in trophoblasts treated with PQR620, a specific mTOR signaling inhibitor, the autophagy and proliferation in trophoblasts were decreased. Studies on human placental explants also confirmed our findings by showing that the expression levels of EZH2 and mTOR were decreased while the autophagy-associated protein level was increased by AD-MSC-derived exosome treatment. In summary, our results suggest that EZH2-dependent mTOR signaling inactivation mediated by AD-MSC-derived exosomes is a prerequisite for autophagy augmentation in hypoxic trophoblasts.

## Introduction

Preeclampsia (PE) is a hypertensive disorder during pregnancy, and is associated with substantial maternal and perinatal complications (Hladunewich et al., 2007; Rana et al., 2019). It is deemed to be one of the main causes of fetal and maternal death and morbidity across the world, and is reported to have a 2–8% incidence among pregnancies, particularly in the developing countries (Tranquilli et al., 2012). Despite the considerable efforts on its study, the etiology of PE has not yet been clear. At present, uteroplacental malperfusion caused by the trophoblast invasion failure and transformation of spiral arteries is considered as an important cause for this disorder (Ridder et al., 2019). In addition, aberrant placental implantation can cause increased oxidative stress and hypoxia, which leads to inflammation and antiangiogenic protein release (Mol et al., 2016). Ischemia and hypoxia in trophoblasts are important pathological manifestations of PE.

Autophagy is a mechanism that maintains homeostasis through degradation of malfunctioned organelles, redundant molecules and invasive pathogens by lysosomes (Kang et al., 2020). Autophagy also plays important roles in intracellular signaling pathways regulating cell proliferation, differentiation, and replicative senescence (Mo et al., 2020). Under stress conditions such as malnourishment, hypoxia and starvation, autophagy would be activated to provide nutrients and energy to the cells (Mizushima and Komatsu, 2011; Ho et al., 2017). Due to the hypoxic and ischemic responses associated with PE, placental trophoblasts in individuals with PE are more reliant on autophagy for survival than normal cells (Nakashima et al., 2019a,b). Autophagy is crucial in many tissues, including that in placenta (Menikdiwela et al., 2020). It protects syncytiotrophoblasts from apoptosis, infection, and inflammation in the human placenta (Zenclussen et al., 2017; Zhang et al., 2019), but many questions remain regarding the exact etiology and precise pathogenic mechanisms in the autophagic flux.

EZH2 is a bona fide histone methyltransferase, methylating histone 3 at lysine 27 (H3K27) and leading to a selective suppression of transcription by changing the chromatin conformation (Aalia Batool and Liu, 2019). Due to its histone methyltransferase activity, epigenetic mechanisms are often cited in explaining the biological consequences following its suppression or overexpression (Huang et al., 2014; Mu et al., 2017). The role of EZH2 in cancer progression and malignancy has been extensively studied in the last decade (Liu et al., 2018; Emran et al., 2019). Moreover, growing evidences demonstrate that downregulation of EZH2 was essential for inducing autophagy and apoptosis in many types of cancer cells (Xue et al., 2019; Liu et al., 2020). However, the exact role EZH2 plays in trophoblastic autophagy has not been fully elucidated.

Mesenchymal stem cells (MSCs) are versatile cells that can be differentiated into various specialized cells including osteoblasts, scleroblasts, chondrocytes, and adipocytes (Ridge et al., 2017). AD-MSCs have similar phenotypic and functional characteristics to other kinds of MSC (Umezawa et al., 2019). An extra beneficial characteristic of MSC is its capability to attenuate inflammation and tissue ischemia (Bianco et al., 2008; Mazini et al., 2020). These characteristics could be favorable for therapeutic placental villi remodeling and for promoting placental development. Many studies have suggested that AD-MSCs can promote angiogenesis through paracrine effects and potentially participate in placental pathologies in the vascular system, including PE as well as fetal growth restriction (MacDonald and Barrett, 2019). However, currently there is no information on the roles of AD-MSCs in the autophagic capacity of extravillous trophoblasts (EVTs).

In this study, the effects of AD-MSC-derived exosomes on the proliferative capacities of the trophoblast cell lines JEG-3 and HTR-8 were studied. In addition to increasing the proliferative capacities, AD-MSC-derived exosomes significantly enhanced autophagy in the trophoblasts under hypoxic conditions. The transcriptome analysis showed considerable downregulation of the enhancer of zeste 2 polycomb repressive complex 2 subunit (EZH2) and mTOR signaling pathway in trophoblasts treated with treated with AD-MSC-derived exosomes; mTOR was putatively recognized as an upstream inhibitor of autophagy under hypoxic conditions.

## Materials and Methods

### Cell Culture

Placentas were obtained from full-term births after a cesarean section (*n* = 3) with parental permission. Every procedure was conducted in accordance with the ethical protocols of The Affiliated Hospital of Qingdao University, China. Amnions were separated from term placentas. Primary AD-MSCs were extracted from term amnion. AD-MSCs were isolated, cultured and characterized as published (Konig et al., 2012; König et al., 2015). All AD-MSCs were used at passages 3–5 in this study. The isolated cells were plated on culture plates in stem cell culture medium (SCCM), which contained Stem Cell Basic Medium (Dakewe Biotech Co., Guangzhou, China) and 5% UltraGRO^TM^ (Helios, United States). An incubator with a temperature of 37°C and 5% CO_2_ was used to culture The primary cells were cultured in an incubator at 37°C in an atmosphere of 5% CO_2_.

The JEG-3 and HTR-8 cells obtained from the Type Culture Collection of China Centre were subjected to culture and then used for experiments. DMEM/F12 containing 10% FBS was used to culture all three trophoblast cell lines in an incubator at 37°C and in an atmosphere of 5% CO_2_. The medium was changed when the confluency reached 50%. The cells were subjected to incubation for a set of time at 37°C, and the humidified atmosphere of the incubator contained 93% N_2,_ 5% CO_2,_ and 2% O_2_ (Invivo2 Hypoxia Workstation, Ruskinn Technology, Leeds, West Yorkshire, United Kingdom). For each experiment, the cells were subjected to culture in triplicate.

### AD-MSC Identification

Flow cytometry (with antibodies obtained from eBioscience, San Diego, CA, United States, including CD34, CD105, CD73, CD90, CD44, CD45, IG1, and HLA-DR) was used to examine the expression of cell markers in AD-MSCs (passage 3); these markers included positive markers (CD44, CD73, CD90, and CD105) and negative markers (CD34, CD45, CD146, IG1, and HLA-DR).

Moreover, AD-MSCs from normal placentas or placentas with severe PE could differentiate into osteoblasts as well as adipocytes; therefore, we assessed their differentiation capability. AD-MSCs cultured in 6-well plates were grown to approximately 70–80% confluency. Then, the AD-MSCs were cultured in differentiation medium (osteogenic or adipogenic) (Gibco, Carlsbad, CA, United States) for 3 weeks. Alizarin red S was used to stain the AD-MSCs to verify osteoblast differentiation; for adipocyte differentiation, oil red O was selected.

### Exosome Isolation

Exosomes were obtained from epidural AD-MSC supernatants by differential centrifugation. The medium was discarded when AD-MSCs reached 70% confluency. Then, the cells were cultured in serum-free DMEM/F12 for another 24 h. The supernatants were collected and then cleared by sequential centrifugation at 15,000 × *g* for 30 min or 3,000 × *g* for 30 min. The supernatants were ultracentrifuged at 120,000 × *g* for 2 h after being filtrated through 0.22-mm filters (Millipore, Billerica, MA, United States). The exosomes were rinsed by sterile PBS and collected several times. The exosome concentrations were determined with a Pierce BCA protein assay kit (Thermo Fisher Scientific).

### Electron Microscopy

In approximately 10 min, almost 50 μl of prepared exosomes were adsorbed and placed onto formvar carbon-coated 300-mesh copper grids. Then, the adsorbed exosomes were dried at room temperature for 30 min and negatively dyed with 3% phosphotungstic acid. Later, by using a transmission electron microscope (Olympus Software Imaging Solutions) at 120.0 kV, the exosomes were examined. Moreover, a digital camera was used to capture images of the exosomes.

### Antibodies and Reagents

We purchased anti-microtubule-associated protein LC3, P62, CD63, and BECN1 (beclin1, an autophagosome initiator) antibodies from R&D Systems (Minneapolis, MI, United States). Cell Signaling Technology (Danvers, MA, United States) provided the following antibodies: anti-EZH2 (#5246), anti-mTOR (#2983), anti-p-mTOR (#5536), anti-S6K1 (#2708), anti-p-S6K1 (#9204), anti-TSG101 (#28405), and anti-Ki67 (#9449). GSK126 (EZH2inhibitor) (10 μM), PQR620 (50 nM) and bafilomycin A1 (Baf A1, an autophagosome-lysosome fusion inhibitor) (100 nM) were purchased from MedChem Express (Monmouth Junction, NJ, United States). EZH2 plasmids were purchased from Shanghai Genechem Co., Ltd.

### Quantitative Real-Time PCR

AD-MSC-derived exosomes (10 μg/ml) was used to treat JEG-3, and HTR-8 cells for 24 h. Then, we isolated the total RNA from the trophoblasts with TRIzol Reagent (Takara, Japan). After that, a reverse transcription kit (Invitrogen) was used to synthesize complementary DNA. Master Mix (Thermo Fisher Scientific) and Gene-specific TaqMan probes (Applied Biosystems) were used to carry out quantitative real-time PCR (RT-PCR) according to the manufacturer’s instructions. The expression of each target gene was normalized to GAPDH expression. We used TaqMan probes for EZH2 (Hs00544830_m1), mTOR (Hs00234508_m1), S6K1 (Hs00356367_m1), and GAPDH (Hs02786624_g1), and conducted three separate reactions for each marker.

### RNA Interference

Short interfering RNA (siRNA) oligonucleotide duplexes targeting EZH2 used in this study were synthesized and purified by RiboBio (Ribobio Co., Guangzhou, China). The sequences are as follows: siEZH2 #1: 5′-GCUGGAAUCAAAGGAUACA-3′; siEZH2 #2: 5′-GCGTTTCTTGTATCGGGAAAT-3′. A nonsense siRNA with no homology to the known genes in human cells was used as negative control: 5′−UUC UCC GAA CGUGUC ACG UTT−3′. Transfections of siRNA in trophoblasts were performed by using Lipofectamine 2,000 (Invitrogen, Carlsbad, CA, United States) according to the manufacturer’s instructions, and the knockdown efficiency was verified 48 h after transfection. All the siRNAs were used at a final concentration of 100 nM.

### Construction of Vectors

To make EZH2 constructs and its mutants, the human EZH2 gene was amplified by PCR and cloned into the SgfI/MluI sites of the pCMV6-Entry vector (Life Technologies).

### Cell Proliferation Analysis

We added trophoblasts to ninety-six-well plates (density: 5,000 cells per well), cultured these cells, and measured trophoblast proliferation daily via CCK-8 assays (Thermo Fisher Scientific, Waltham, MA, United States). We added CCK-8 reagent to each well, and cultured the trophoblasts for another 1.5 h. Then, colorimetric assays were performed by measuring the absorbance [optical density (OD) value] of each well in a microplate reader (wavelength: 450 nm). The growth curves were ascertained in three separate experiments.

### EdU Assay

According to the manufacturer’s instructions (Guangzhou RiboBio, Guangzhou, China), 5-Ethynyl-2’-deoxyuridine (EdU) assays were conducted by using a Cell-Light EdU *in vitro* flow cytometry kit. In brief, in a 6-well plate cells were cultured overnight, with a 20-min incubation with EdU followed. Then, they were fixed with 70% ethanol at −20°C overnight and washed twice with PBS. Later, the cells were stained with a FITC-conjugated secondary antibody at ambient temperature for 1 h and denatured in 2 N HCl for 45 min. Moreover, with 40 g/ml RNase A and 200 g/ml PI, the cells were incubated for 30 min and finally analyzed by flow cytometry.

### Western Blotting

Trophoblasts were lysed on ice for 12 min with RIPA buffer (Sigma, St. Louis, MO, United States). After centrifugation at 12,000 × *g*, the cell lysates were treated with LDS sample buffer. SDS-PAGE was used to separate the protein mixtures, which were then electro-transferred to a polyvinylidene fluoride (PVDF) membrane (Bio-Rad, Hercules, CA, United States). Next, 5% skim milk was used to block the membrane.

Subsequently, primary rabbit monoclonal antibodies against human LC3, BECN1, P62, EZH2, mTOR, p-mTOR, S6K1, and p-S6K1 (1:1,000 dilution) or β-actin (same dilution; Proteintech, Chicago, IL, United States) were incubated with the blocked membranes. Then, secondary antibodies were incubated with the membranes (1:1,000; CST, Danvers, MA, United States). The protein-antibody complexes were detected and quantified by using a chemiluminescence detection system (Bio-Rad, Hercules, CA, United States).

### Placental Explant Culture

All placentas were collected after operation, treated within 30 min and closely examined for any visible abnormalities. After thorough rinsing with PBS 3 times to remove the maternal blood, the placental villous tissues were chopped into 8-mm^3^ pieces (2 mm × 2 mm × 2 mm). DMEM/F12 (4 ml per well) with 1% penicillin/streptomycin and amphotericin B (Gibco, Carlsbad, CA, United States) was used to culture the placental explants in six-well dishes (Corning) in a hypoxic incubator for 48 h at 37°C in an atmosphere of 2% oxygen. After AD-MSC-derived exosomes treatment for 24 h, PBS was used to rinse the explants; after that, they were frozen in liquid nitrogen.

### Immunohistochemistry

Paraformaldehyde (4%) was used to fix the human term placental explants for 60 min. We embedded the tissues in paraffin, sliced them into 4-μm sections, and deparaffinized them. Then, the slides were boiled in 6.0 pH sodium citrate buffer (10 mM) for 7 min at 120°C for antigen retrieval. Hydrogen peroxide was used to block endogenous peroxidase for 10 min. We subsequently washed the slides three times for 5 min each with TBS (containing 0.05% Tween 20) (TBS/T; Merck; Darmstadt, Germany); later, these slides were incubated with monoclonal anti-EZH2 antibodies (1:200) for 12 h at 4°C. Diluted biotin-labeled secondary antibodies were incubated with these sections for 20 min at 37°C. We visualized the target proteins via fresh DAB solution and used hematoxylin as a tissue counterstain. Using an optical microscope (Olympus FV500, Tokyo, Japan), the expression of the target proteins was assessed by two observers independently. The staining area and intensity in five different random regions (200× magnification) were analyzed with Image-Pro Plus 5.1 to assess the protein expression levels.

### Immunofluorescence

Trophoblasts were cultured with or without purified AD-MSC-derived exosomes for 24 h (10 μg/ml). Then, all the cells were gathered and separated, and later for 60 min, they were fixed with 4% paraformaldehyde. The fixed cells were cut into 4-μm sections and embedded in paraffin. Later, they were washed in PBS for three times and blocked with 10% goat serum for 1 h. After that in 0.2% Triton X-100 the sections were washed twice. Next, they were incubated with primary antibodies (anti-LC3-II and anti-tubulin were purchased from Abcam, Cambridge, MA, United States), secondary antibodies (Invitrogen) and DAPI (Guangzhou RiboBio, Guangzhou, China). Using a fluorescence microscope, images were captured.

### Statistical Analysis

One-way ANOVA or two-tailed Student’s *t*-test was adopted to carry out statistical analysis; the data are reported as the mean ± standard deviation (SD) from more than three experiments performed independently. If the *P* value was less than 0.05, it indicated a statically significant difference.

## Results

### Proliferation and Autophagy Were Promoted in Trophoblasts by AD-MSCs Under Hypoxic Conditions

**FIGURE 1:**
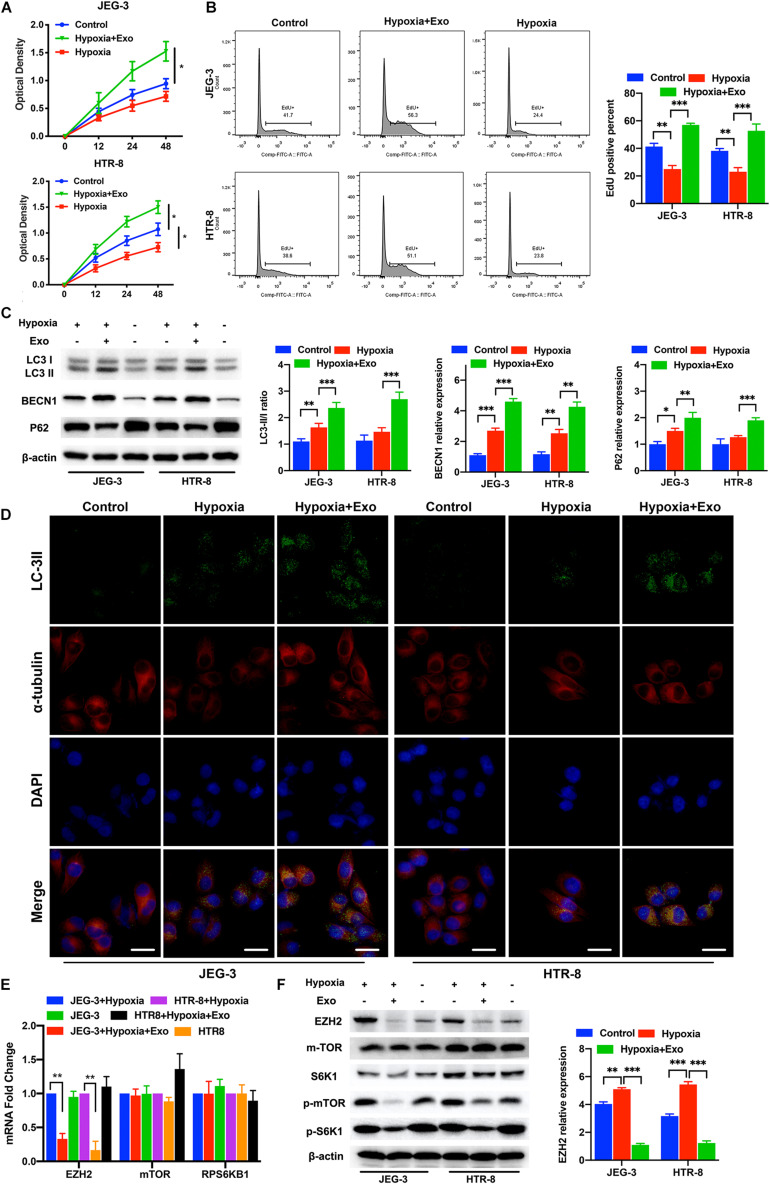
AD-MSC-derived exosomes promote the autophagy and proliferation of trophoblasts in hypoxia condition. **(A)** Representative CCK-8 assay results for JEG-3 and HTR-8 cells are shown. Trophoblast cells were treated with AD-MSC exosomes under hypoxic conditions. **(B)** Representative EdU assay results for trophoblasts are shown. The histogram of EdU positive trophoblasts that treated with AD-MSC exosomes under hypoxic conditions was shown. **(C)** Whole cell lysates from trophoblast cells were subjected to western blotting to analyze and quantificate LC3, BECN1 and p62 levels. β-actin was included as a loading control. **(D)** Immunofluorescence analysis showed autophagosomes in the cytoplasm of trophoblast cells treated with AD-MSC exosomes under hypoxic conditions (scale bar, 25 μm). **(E)** Significant decreases in EZH2, mTOR and S6K1 mRNA levels were found in JEG-3 and HTR-8 cells treated with AD-MSC exosomes by qRT-PCR. **(F)** Whole cell lysates from trophoblast cells were subjected to western blotting to analyze EZH2, mTOR, S6K1, p-mTOR and p-S6K1 levels and quantificate EZH2 expression. β-actin was included as a loading control. *P < 0.05, **P < 0.01, ***P < 0.001.

First, we isolated AD-MSCs from healthy placentas, and identified their multidirectional differentiation ability and surface marker expression (Supplementary Figures 1A,B). Then, we identified the exosomes isolated from AD-MSCs and observed the exosomes endocytosis in trophoblasts (Supplementary Figure 1C). Next, we used the CCK-8 assays, EdU assays, immunofluorescence and Western blotting to examine the proliferation and autophagy of trophoblasts treated with or without AD-MSC-derived exosomes under hypoxic conditions (Figures 1A–D). The AD-MSC-derived exosome-treated trophoblasts exhibited significantly higher proliferation rates than the untreated cells under hypoxic conditions (Figures 1A,B, all *P* < 0.001). To further verify the autophagy-promoting effect of AD-MSC-derived exosomes on trophoblasts, we examined the LC3-II/LC3-I ratio, BECN1 and P62 levels in trophoblasts treated with AD-MSC-derived exosomes by western blotting. The AD-MSC-derived exosome-treated trophoblasts exhibited significantly higher levels of LC3 and BECN1 than the untreated cells (all *P* < 0.05), and the P62 levels of the two trophoblast cell lines was decreased after treatment with AD-MSC-derived exosomes (Figure 1C). According to the immunofluorescence assay results, AD-MSC-derived exosomes increased the staining intensity and area of punctate LC3-II in trophoblasts after 24 h of exposure to the hypoxic culture system (Figure 1D). These results indicated that the AD-MSCs promoted trophoblast proliferation and autophagy under hypoxic conditions by secreting exosomes.

### The EZH2 Expression Was Underregulated in Trophoblasts by AD-MSC-Derived Exosome Treatment Under Hypoxic Conditions, Also Accompanied by the Inhibition of mTOR Pathway

To better understand the changes in trophoblasts that occurred after AD-MSC-derived exosome treatment, we compared the EZH2 levels in the two trophoblast cell lines cultured for 48 h with or without AD-MSC-derived exosomes under hypoxic conditions. The mRNA levels of EZH2 significantly decreased in trophoblasts treated with AD-MSC-derived exosomes (Figure 1E). Many researches have revealed that the mTOR pathway plays a vital role in intracellular metabolism regulation and could regulated by EZH2, suggesting that it is involved in regulating autophagy (Wei et al., 2016; Hu, 2019). Therefore, we examined whether the mTOR pathway could be regulated by the AD-MSC-derived exosomes. Our results revealed that AD-MSC-derived exosome-treated trophoblasts (48 h treatment) exhibited lower mTOR and RPS6KB1 mRNA levels than untreated trophoblasts (Figure 1E). In consistence with the data of mRNA levels, the EZH2, p-mTOR and p-S6K1 protein levels were significantly decreased in AD-MSC-derived exosome-treated trophoblasts compared to the control trophoblasts (Figure 1F and Supplementary Figure 2B). Our results suggested that the mTOR signaling pathway could be inhibited by AD-MSC-derived exosome treatment in trophoblasts under hypoxic conditions, which is also accompanied by decreased EZH2 expression.

### The Downregulation of EZH2 Induced the Increase of Trophoblast Autophagy and mTOR Pathway Inhibition Under Hypoxic Conditions

**FIGURE 2:**
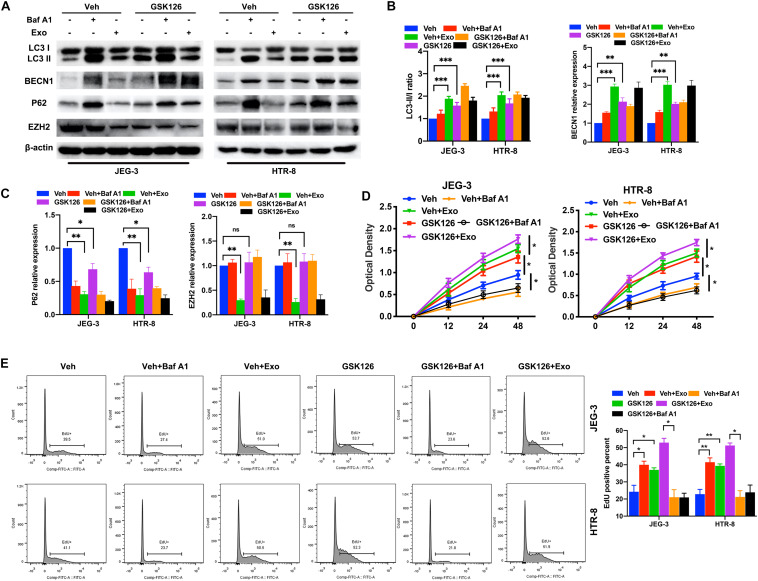
The inhibition of EZH2 induced the increase of trophoblast autophagy and inhibition of mTOR pathway under hypoxic conditions. **(A–C)** The protein expression level of LC3, BECN1 and P62 levels were examined in JEG-3 and HTR-8 cells treated with GSK126, AD-MSC exosomes or Baf A1 for 24 h by western blot. The protein expression levels were quantified by densitometry. **(D)** Cell proliferation was evaluated by a CCK-8 assay. The trophoblast cell lines were treated with GSK126 or Baf A1 and with or without AD-MSC exosomes under hypoxic conditions. **(E)** The EdU positive percent of trophoblast cell lines were tested under hypoxic conditions by flow cytometry. **P < 0.01, *P < 0.05, ***P < 0.001.

**FIGURE 3:**
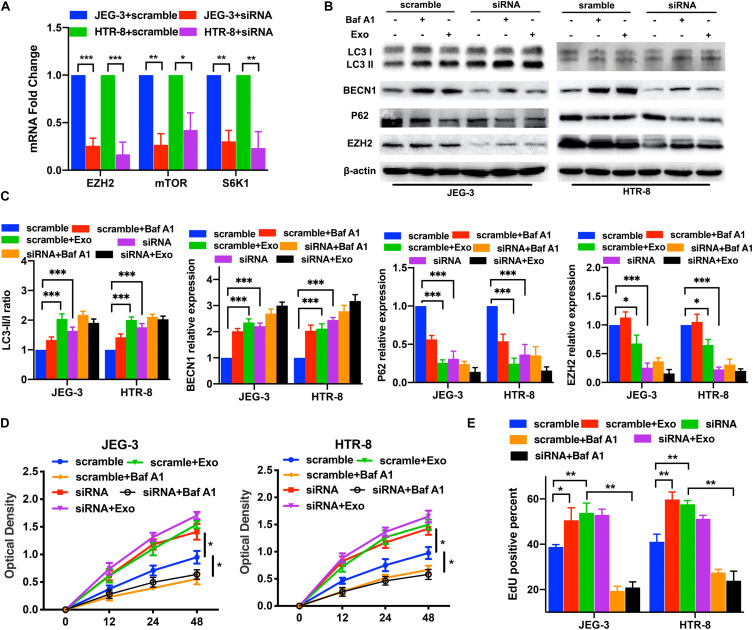
The knockdown of EZH2 induced the increase of trophoblast autophagy and mTOR pathway inhibition under hypoxic conditions. **(A)** Significant decreases in EZH2, mTOR and S6K1 mRNA levels were tested in JEG-3 and HTR-8 cells transfected with EZH2 siRNA by qRT-PCR. **(B,C)** The trophoblast cell lines were transfected with a EZH2 siRNA under hypoxic conditions, and BECN1, P62 and LC3 expression levels were tested by western blotting analysis. Empty vector (scramble) cells served as controls. **(D,E)** Cell proliferation was evaluated by a CCK-8 assay and EdU flow cytometry. The two trophoblast cell lines were transfected with EZH2 siRNA with or without AD-MSC exosomes treatment under hypoxic conditions. *P < 0.05, **P < 0.01, ***P < 0.001.

After confirming that AD-MSC-derived exosomes activated autophagy and inhibited the EZH2 expression in trophoblasts, we investigated whether EZH2 could regulate the trophoblast autophagy. Trophoblasts were treated with the specific EZH2 inhibitor GSK126 and autophagy inhibitor Baf A1. The results confirmed the inhibiting effective concentration of GSK126 on the H3k27me3 levels in trophoblasts (Supplementary Figure 1D), and 10 μM GSK126 was used in further study. LC3-II/LC3-I ratio and BECN1 levels in trophoblasts were significantly increased, P62 levels were decreased in the presence of GSK126 (10 μM), whether with or without AD-MSC-derived exosome. Meanwhile Baf A1 treatment increased LC3-II levels because of blocking autophagic flux (Figures 2A–C). In consistence with the data of LC3-II/LC3-I ratio in Western blotting, GSK126 and ADSCs exosomes promote the LC3-II levels in trophoblasts by Immunofluorescence assays (Supplementary Figure 3A). In addition, we discovered that p-mTOR, p-RPS6KB1 and P62 protein levels decreased; and the LC3-II/LC3-I ratio and BECN1 level increased in the trophoblasts in the presence of GSK126 than in its absence. We studied the same parameters in cells treated with the EZH2 siRNA (Figures 3A–C and Supplementary Figure 3B). In addition, we examined the proliferation of trophoblasts treated with EZH2 inhibitor or transfected with EZH2 siRNA in the presence of AD-MSC-derived exosome or Baf A1, and found that the trophoblast proliferation had the same change curve as the autophagy activity (Figures 2D,E, 3D,E).

### The EZH2 Overexpression in Trophoblasts Inhibited Autophagy and Activated mTOR Pathway Under Hypoxic Conditions

**FIGURE 4:**
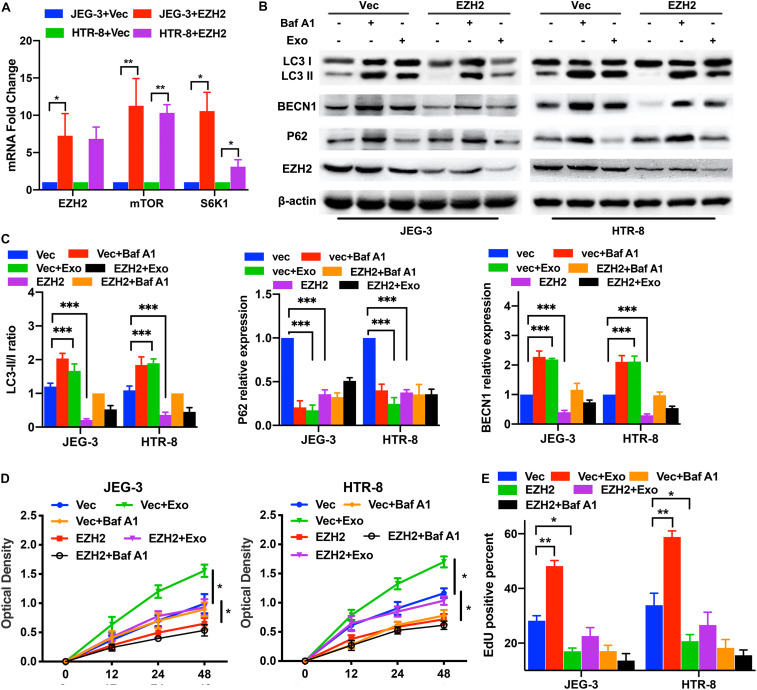
EZH2 overexpression decreased trophoblast autophagy and proliferation through mTOR signaling pathway. **(A)** Significant increases in EZH2, mTOR and S6K1 mRNA were found in JEG-3 and HTR-8 cells transfected with EZH2 overexpression plasmid (EZH2) compared to empty vector (Vec)-transfected cells under hypoxic conditions by qRT-PCR. **(B,C)** The two trophoblast cell lines were transfected with EZH2 overexpression plasmid under hypoxic conditions, and BECN1, P62 and LC3 protein levels were tested by western blotting analysis. **(D,E)** Cell proliferation was evaluated by a CCK-8 assay and EdU flow cytometry. The trophoblast cell lines were transfected with a EZH2 overexpression plasmid or empty vector under hypoxic conditions. *P < 0.05, **P < 0.01, ***P < 0.001.

First, we confirmed that the overexpression plasmids of EZH2 could elevate the EZH2 mRNA levels in trophoblasts (Figure 4A). To determine whether the EZH2 plasmids present in trophoblasts were responsible for the observed effects, we examined the mTOR signaling and autophagy-related proteins in trophoblasts treated with EZH2 plasmids and AD-MSC-derived exosomes using Western blotting (Figures 4B,C and Supplementary Figures 2A, 3C). The results showed that the EZH2 overexpression attenuated the AD-MSC-derived exosome-induced inhibition of mTOR signaling and decreased autophagy in trophoblasts. In addition, we examined the proliferation of trophoblasts treated with EZH2 overexpression plasmids in the presence of AD-MSC-derived exosome or Baf A1, and found that the trophoblast proliferation had the same change curve as the autophagy activity (Figures 4D,E). Thus, it revealed that EZH2 acted as a down-regulator of AD-MSC-derived exosome-induced autophagy and that mTOR signaling may act as an downstream autophagy inhibitor regulated by EZH2 in trophoblasts.

### EZH2 Regulate Trophoblast Autophagy and Proliferation Through mTOR Signaling Pathway

**FIGURE 5:**
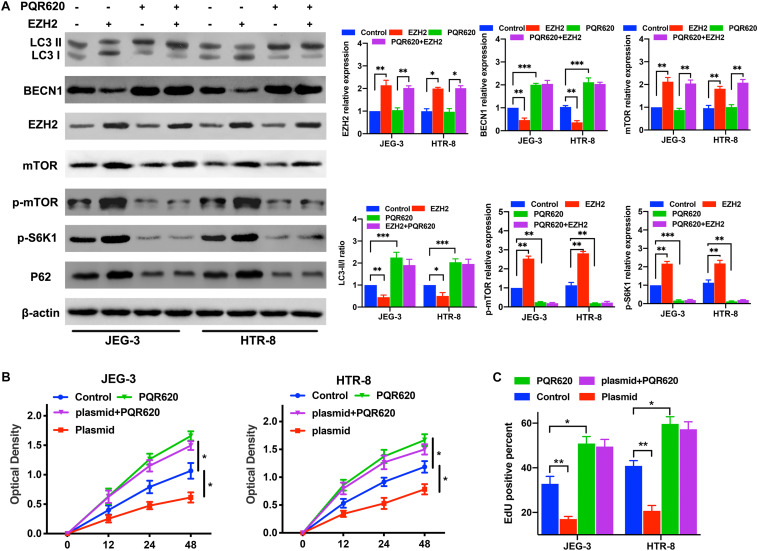
EZH2 regulate trophoblast autophagy and proliferation through mTOR signaling pathway. **(A)** The levels of EZH2, p-mTOR, p-S6K1, and autophagy associated proteins were examined in JEG-3 and HTR-8 cells transfected with EZH2 overexpression plasmid and treated with m-TOR inhibitor PQR620 under hypoxic conditions by western blot. **(B,C)** Cell proliferation was evaluated by a CCK-8 assay and EdU flow cytometry. The trophoblast cell lines were treated with EZH2 overexpression plasmid and m-TOR inhibitor PQR620 under hypoxic conditions. *P < 0.05, **P < 0.01, ***P < 0.001.

To assess whether EZH2 mediated trophoblasts autophagy inhibition through the mTOR signaling pathway, the trophoblasts were transfected with EZH2-overexpression plasmids and treated with PQR620, a highly potent and selective mTOR inhibitor. Then, the expression levels of EZH2, mTOR and autophagy associated proteins in trophoblasts were evaluated by Western blotting. The Western blotting results confirmed that EZH2 overexpression in trophoblasts activated mTOR signaling and inhibited the autophagy in trophoblasts, and mTOR inhibitor attenuated the EZH2-mediated autophagy inhibition (Figure 5A). Moreover, the proliferation assays suggested that EZH2 overexpression and mTOR signaling activation inhibited the trophoblast autophagy and proliferation, and PQR620 treatment reversed such inhibition (Figures 5B,C). These results indicates that EZH2 in trophoblasts may regulate their autophagy through the mTOR signaling pathway.

### AD-MSC-Derived Exosome-Mediated EZH2 Inhibition Increased Autophagy in Placental Explants

**FIGURE 6:**
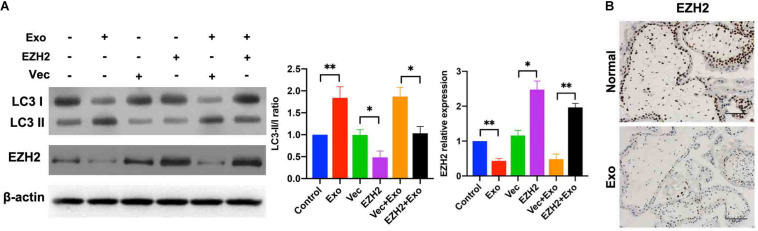
AD-MSC-mediated EZH2 activation increased autophagy in placental explants under hypoxic conditions. **(A)** Placental explants were treated with AD-MSC exosomes transfected with EZH2 overexpression plasmids or Vec under hypoxic conditions, and EZH2, LC3 expression levels were tested by western blotting analysis. **(B)** Placental explants were treated with AD-MSC exosomes under hypoxic conditions, and EZH2 were tested by immunohistochemistry. Untreated placental explants served as controls (scale bar, 50 μm). *P < 0.05, **P < 0.01.

Next, the data from trophoblasts were compared with those from placental explant cultures, which are *in vivo* models of trophoblasts in which villous trophoblasts remain in a natural environment and under more physiologically relevant conditions than the traditional cell culture. The human placental explants were incubated in DMEM/F12 under hypoxic condition and then cultured in a medium containing AD-MSC-derived exosomes transfected with or without EZH2 overexpression plasmids for 48 h. The Western blotting assays were used to determine the levels of EZH2 and autophagy associated proteins in the placental explants. While autophagy levels were significantly increased in the AD-MSC-derived exosomes-treated placental explants, and plasmids transfection attenuated the increase. Moreover, compared to the control explants, the EZH2 levels showed significant decrease in AD-MSC exosomes treated explants, and the plasmids transfection could elevate the EZH2 expression in explants (Figure 6A). Additionally, the immunohistochemical analysis showed a reduced EZH2 intensity in the villous cytotrophoblasts in AD-MSC-derived exosome-treated placental explants (Figure 6B).

Taken together, these data suggest that AD-MSC-derived exosomes inhibit the EZH2 expression and mTOR signaling pathway, and increase autophagic activity in cultured placental explants.

## Discussion

The amnion is the innermost avascular layer of the embryonic membranes and is an important source of promising cells that have therapeutic value. It has been used to improve a variety of medical conditions such as ophthalmology, skin injuries, and premature ovarian insufficiency. Its therapeutic effects are attributed to its inflammation-counteracting and immunomodulatory properties. Although the exact mechanisms are not clear yet, factors secreted by AD-MSCs are suggested to be the great contributors. In spite of the fact that the pathological mechanism of PE is illusive, dysfunction and hypoxia in trophoblasts were thought to be associated with preeclamptic placentas (Nakashima et al., 2017a,b). In this study, we provide evidences that AD-MSC-derived exosome-mediated inhibition of EZH2 expression and mTOR signaling pathway is the main mechanisms of autophagic activity regulation in human villous trophoblasts and that it improves trophoblast survival in hypoxia conditions.

Autophagy was once deemed to occur in cytoplasm; however, recently, more and more evidences suggest that nuclear machineries (transcription factors, histone modification, microRNAs, etc.) are also involved in autophagy regulation (Boban and Foisner, 2016). Among them, epigenetic autophagy regulation has been given lots of attention. The epigenetic machinery can both modulate autophagy-related genes directly and affect some signal transduction genes that can in turn regulate autophagy, thereby influencing their transcription and autophagy subsequently (Shin et al., 2016). The methylation of DNA and modifications of histone were considered to be involved in autophagy regulation recently (Fullgrabe et al., 2014). The overexpression of EZH2 has been observed in a variety of cancers, and was correlated to cancer progression, metastasis and poor prognosis in various cancer types (Gan et al., 2018). In recent time, it is found that EZH2 might make a great contribution in autophagy (Shin et al., 2016). Particularly, downregulation of EZH2 was found as an epigenetic modulator of autophagy by regulating the mTOR pathway in the colorectal carcinoma (Wei et al., 2016). However, the mechanism involved in the epigenetic regulation of autophagy in trophoblasts is far from being fully known.

The mechanistic target of rapamycin (formerly mTOR, mammalian target of rapamycin) is a serine/threonine protein kinase that is evolutionarily conserved, playing a central role in regulating the cell growth, proliferation and survival, according to the condition of nutrition, signals of stress and growth factors. It is a crucial controller of fundamental biological processes including lipid and glucose metabolism, autophagy, apoptosis, etc. Many researches have identified mTOR as a vital autophagy regulator, and deregulations of the mTOR pathway have been found to be involved in various pathological failures. On a molecular basis, autophagy regulates several signaling pathways that determines the death or continued survival of cells; nevertheless, the relationship between autophagy, EZH2 and mTOR pathways in trophoblasts is still undecided. The fate of trophoblasts, which were involved in hypertensive diseases during pregnancy, was affected by the autophagy regulation by AD-MSC-derived exosomes. In this study, the EZH2 expression was found to be reduced in trophoblasts treated with AD-MSC-derived exosomes, accompanied by the inactivation of the mTOR signaling pathway. These findings confirmed the curative effects of AD-MSC secretions and provided a novel insight into potential PE therapies.

In summary, it is revealed in our study that AD-MSCs promote the trophoblast proliferation and autophagy under hypoxic conditions *in vitro*, partially due to inactivation of mTOR signaling induced by the EZH2 downregulation. However, it remains unclear how AD-MSCs influence other types of placenta-based cells *in vitro* or even *in vivo*; thus, further studies are needed on whether AD-MSCs affect them similarly. More studies on how EZH2 regulates autophagy will be important for a thorough understanding of the PE pathogenesis.

## Data Availability Statement

The raw data supporting the conclusions of this article will be made available by the authors, without undue reservation.

## Ethics Statement

The studies involving human participants were reviewed and approved by The Affiliated Hospital of Qingdao University. The patients/participants provided their written informed consent to participate in this study.

## Author Contributions

YC, YH, and YY conceived and designed the experiments. WC, YoL, YZ, JuZ, and HZ performed the experiments. WP, LX, JiZ, NZ, GG, and XW collected the samples. LL, JL, RH, CL, and KY analyzed the data. YC, HZ, and YaL wrote the manuscript. All authors read and approved the final manuscript.

## Conflict of Interest

The authors declare that the research was conducted in the absence of any commercial or financial relationships that could be construed as a potential conflict of interest.

## Supplementary Material

Supplementary Figure 1 Characterization of primary AD-MSCs derived from human placental tissues. **(A)** Representative photomicrographs of primary human AD-MSCs before confluence at passage 3. The cells were examined for osteogenic and adipogenic differentiation. Scale bar = 20 μm. **(B)** The purity of the isolated AD-MSCs and PE-AD-MSCs was examined by flow cytometry; AD-MSCs express CD44, CD73, CD90, and CD105, but lack CD34, CD45, CD146, IG1, and HLA-DR expression. **(C)** AD-MSC exosomes were visualized by electron microscopy (×30,000). Western blot examined the expression of exosomes marker TSG101 and CD63 in isolated AD-MSC exosomes. Typical imagines of internalizated exosomes derived from epidural AD-MSCs by trophoblasts JEG-3 at 4 h. Fluorescence microscopy images showing the internalization of exosomes by JEG-3 cells. Blue: Nucleus stained with DAPI. Red: PKH26-labeled exosomes. Scale bar: 50 μm. **(D)** The protein levels of H3K27me3, H3K4me3, H3 and EZH2 in JEG-3 cell treated with GSK126 concentration gradient for 6 h.

Supplementary Figure 2 Effect of EZH2 siRNA, EZH2 inhibitor and overexpression plasmids on the EZH2 and mTOR pathways proteins expression of trophoblasts by western blot. **(A)** Protein expression of EZH2, mTOR, S6K1, p-mTOR, and p-S6K1 was examined in JEG-3 and HTR-8 cells treated with EZH2 siRNA, EZH2 inhibitor or overexpression plasmids by western blotting. **(B)** The levels of mTOR, S6K1, p-mTOR, and p-S6K1 in Figure 1F were quantified by densitometry. Data represent mean ± SD (error bars) of three independent experiments. ^∗∗^*P* < 0.01, ^∗∗∗^*P* < 0.001.

Supplementary Figure 3 Levels of LC3-II in EZH2 siRNA, inhibitor and overexpression plasmids treated trophoblasts with or without ADSCs/Baf A1 by Immunofluorescence assays. **(A)** Expression of LC3-II in trophoblasts treated with GSK126, ADSCs exosomes and Baf A1 was examined by Immunofluorescence assays, and typical images were shown. **(B)** Expression of LC3-II in EZH2 siRNA transfected trophoblasts treated with or without ADSCs exosomes/Baf A1 was examined by Immunofluorescence assays, and typical images were shown. **(C)** Expression of LC3-II in EZH2 overexpression plasmids transfected trophoblasts treated with or without ADSCs exosomes/Baf A1 was examined by Immunofluorescence assays, and typical images were shown (scale bar, 25 μm).
