# Supplementary figures and images for: Amnion-Derived Mesenchymal Stem Cell Exosomes-Mediated Autophagy Promotes the Survival of Trophoblasts Under Hypoxia Through mTOR Pathway by the Downregulation of EZH2
Source: Front Cell Dev Biol. 2020 Nov 11;8:545852. doi: 10.3389/fcell.2020.545852 (PMC7693549; doi:10.3389/fcell.2020.545852)

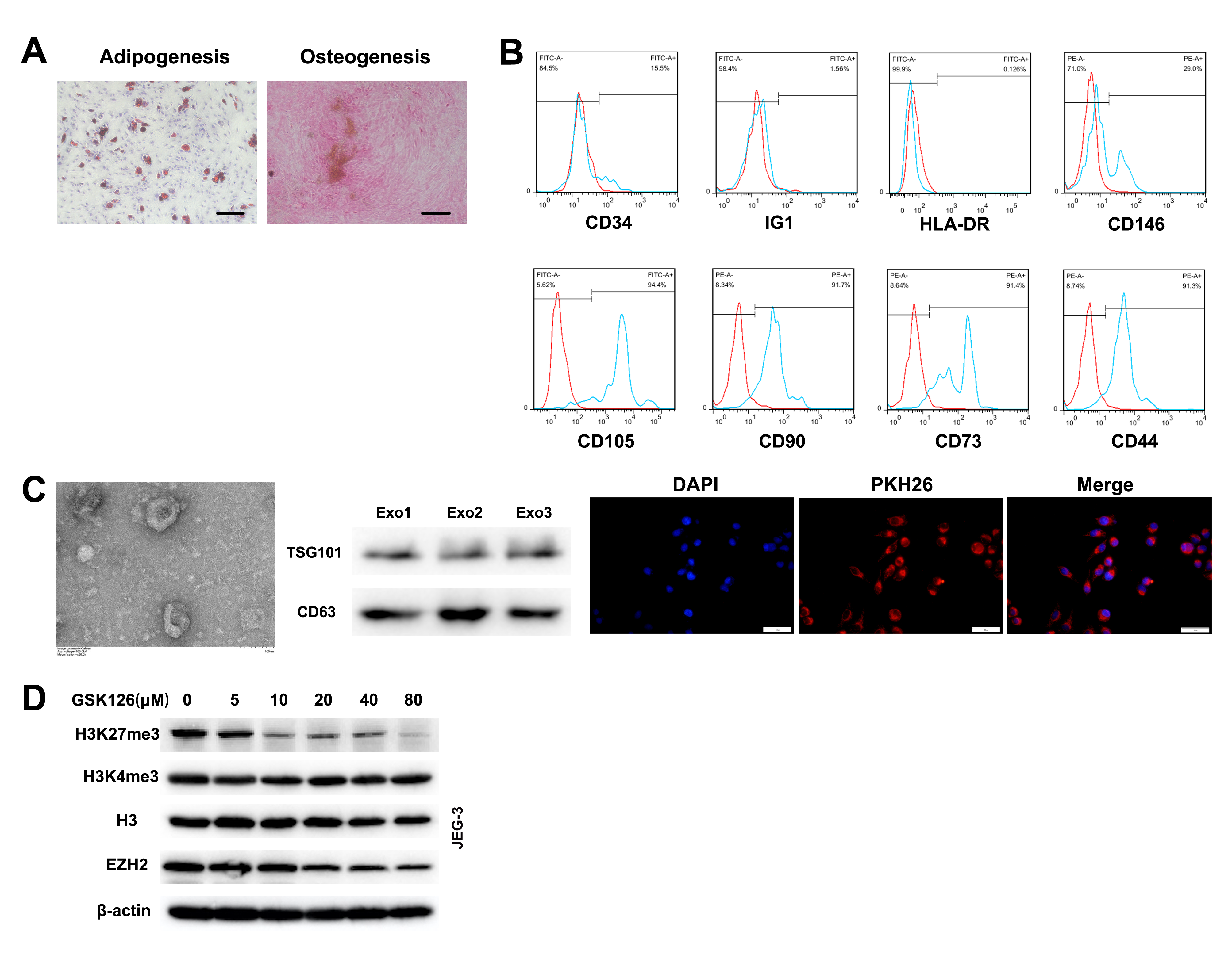

Supplement: Supplementary Figure 1 — Characterization of primary AD-MSCs derived from human placental tissues. (A) Representative photomicrographs of primary human AD-MSCs before confluence at passage 3. The cells were examined for osteogenic and adipogenic differentiation. Scale bar = 20 μm. (B) The purity of the isolated AD-MSCs and PE-AD-MSCs was examined by flow cytometry; AD-MSCs express CD44, CD73, CD90, and CD105, but lack CD34, CD45, CD146, IG1, and HLA-DR expression. (C) AD-MSC exosomes were visualized by electron microscopy (×30,000). Western blot examined the expression of exosomes marker TSG101 and CD63 in isolated AD-MSC exosomes. Typical imagines of internalizated exosomes derived from epidural AD-MSCs by trophoblasts JEG-3 at 4 h. Fluorescence microscopy images showing the internalization of exosomes by JEG-3 cells. Blue: Nucleus stained with DAPI. Red: PKH26-labeled exosomes. Scale bar: 50 μm. (D) The protein levels of H3K27me3, H3K4me3, H3 and EZH2 in JEG-3 cell treated with GSK126 concentration gradient for 6 h. [file Image_1.TIF]

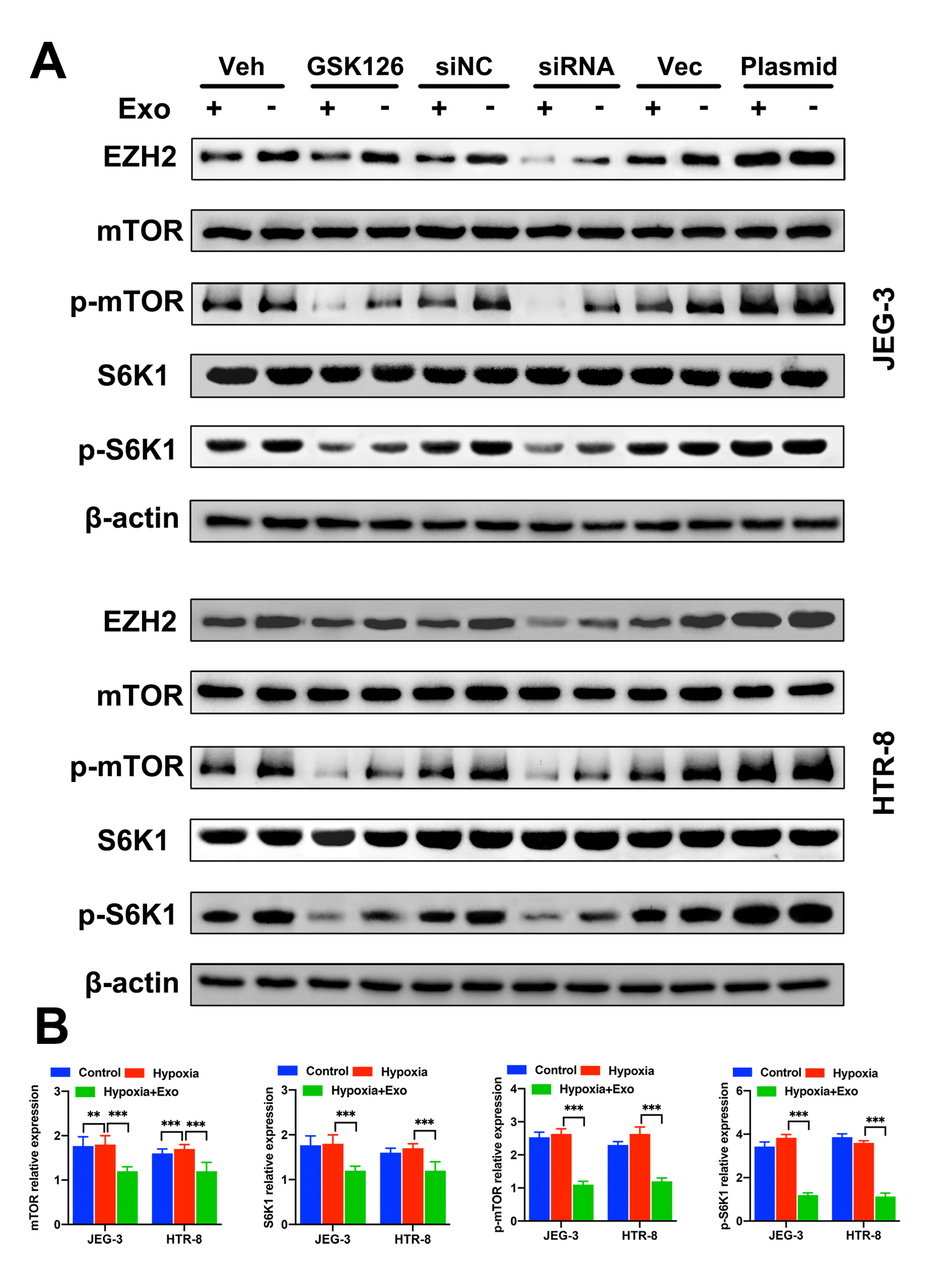

Supplement: Supplementary Figure 2 — Effect of EZH2 siRNA, EZH2 inhibitor and overexpression plasmids on the EZH2 and mTOR pathways proteins expression of trophoblasts by western blot. (A) Protein expression of EZH2, mTOR, S6K1, p-mTOR, and p-S6K1 was examined in JEG-3 and HTR-8 cells treated with EZH2 siRNA, EZH2 inhibitor or overexpression plasmids by western blotting. (B) The levels of mTOR, S6K1, p-mTOR, and p-S6K1 in Figure 1F were quantified by densitometry. Data represent mean ± SD (error bars) of three independent experiments. ∗∗P < 0.01, ∗∗∗P < 0.001. [file Image_2.TIF]

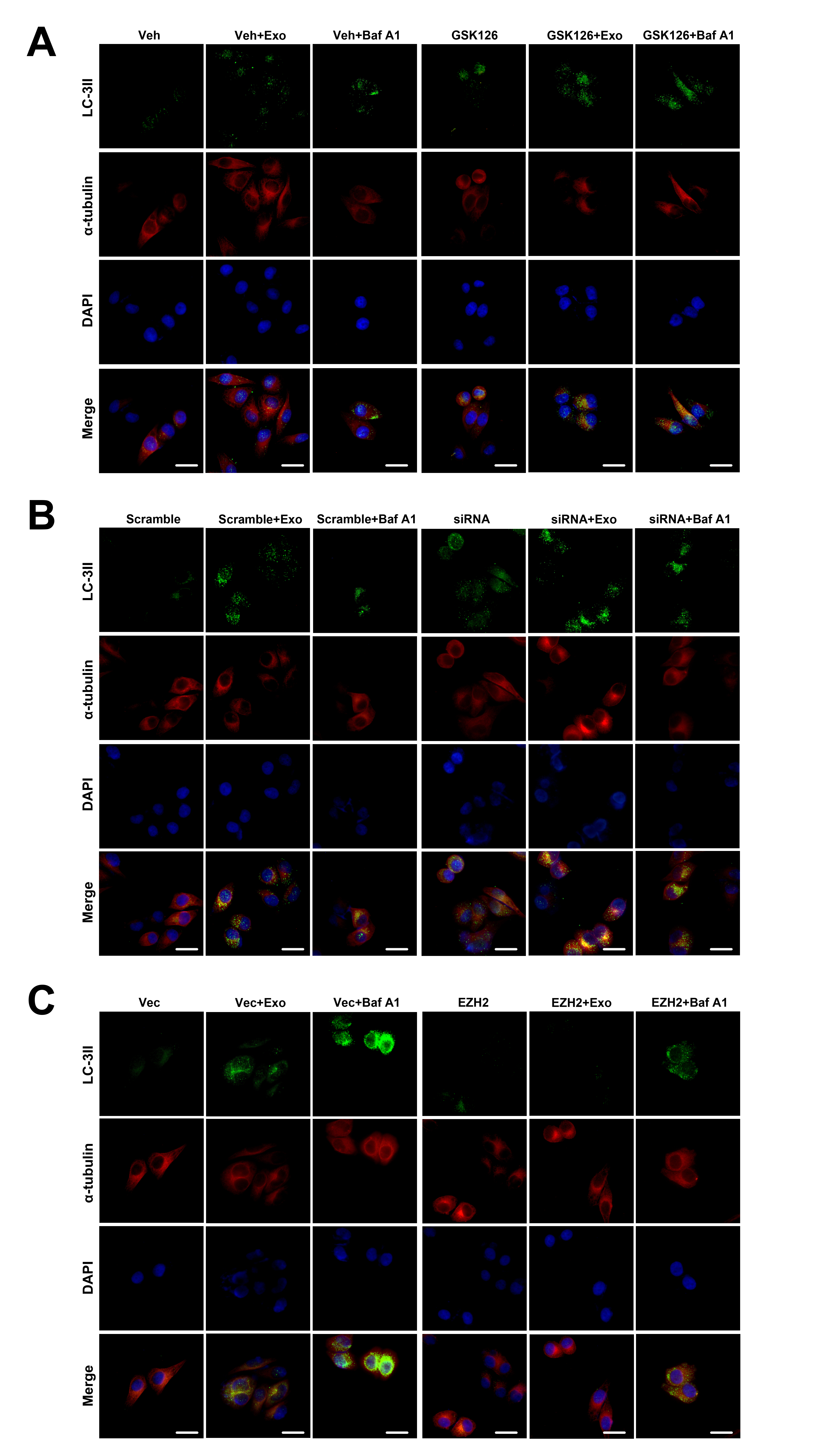

Supplement: Supplementary Figure 3 — Levels of LC3-II in EZH2 siRNA, inhibitor and overexpression plasmids treated trophoblasts with or without ADSCs/Baf A1 by Immunofluorescence assays. (A) Expression of LC3-II in trophoblasts treated with GSK126, ADSCs exosomes and Baf A1 was examined by Immunofluorescence assays, and typical images were shown. (B) Expression of LC3-II in EZH2 siRNA transfected trophoblasts treated with or without ADSCs exosomes/Baf A1 was examined by Immunofluorescence assays, and typical images were shown. (C) Expression of LC3-II in EZH2 overexpression plasmids transfected trophoblasts treated with or without ADSCs exosomes/Baf A1 was examined by Immunofluorescence assays, and typical images were shown (scale bar, 25 μm). [file Image_3.TIF]
